# Autoimmune Disease-Related Dry Eye Diseases and Their Placement Under the Revised Classification Systems: An Update

**DOI:** 10.7759/cureus.50276

**Published:** 2023-12-10

**Authors:** Alara Kılıççıoğlu, Deniz Oncel, Ali Riza Cenk Celebi

**Affiliations:** 1 Neurology, Szeged University, Szeged, HUN; 2 Ophthalmology, Acibadem University, Istanbul, TUR; 3 Ophthalmology, Stritch School of Medicine, Loyola University Chicago, Chicago, USA

**Keywords:** treatment of ded, autoimmune related ded, keratoconjunctivitis sicca, autoimmune disease, dry eye disease

## Abstract

Dry eye disease (DED) is a chronic and progressive disorder involving the ocular surface, characterized by disturbances in tear film composition, instability of the tear film, and inflammation of the ocular surface. There are two forms of DED: aqueous-deficient dry eye (ADDE) and evaporative dry eye (EDE). Autoimmune diseases are systemic disorders involving multiple organs, including the eyes, and have a significant impact on DED. There have been multiple studies demonstrating the relation between autoimmune diseases and DED. This article reviews the current knowledge regarding the epidemiological characteristics, pathogenesis, and treatments of autoimmune disease-related DED.

## Introduction and background

Dry eye disease (DED) or keratoconjunctivitis sicca (KCS) is considered an ocular surface autoimmune disorder [1]. According to the latest report published by The Tear Film and Ocular Surface Society (TFOS), two forms of DED are recognized: aqueous-deficient dry eye (ADDE) and evaporative dry eye (EDE) [2]. In ADDE, tear hyperosmolarity occurs due to decreased lacrimal secretion, despite normal evaporation from the eye. Conversely, in EDE, tear hyperosmolarity is attributed to the excessive evaporation of the exposed tear film, even with a properly functioning lacrimal gland. 

The TFOS Dry Eye Workshop (DEWS) II defines DED as a chronic, multifactorial, and progressive disorder involving decreased tear production, excessive tear evaporation, loss of homeostasis of the tear film, and accompanied by ocular symptoms, in which tear instability and hyperosmolarity, ocular surface inflammation and damage, and neurosensory abnormalities play etiological roles [3-8]. The coordination of these activities depends on an intricate interaction of inputs from the neural, humoral, endocrine, vascular, and immune systems [9]. Environmental elements, an individual's systemic well-being, dietary and social factors, as well as medications, can exert influence on this delicate equilibrium [9,10]. Research on the incidence of dry eye reveals considerable disparities among various regions, with prevalence rates fluctuating from 5% to 50% globally [4,11]. Clayton et al. showed that the incidence of dry eye in recent years has notably risen due to factors such as prolonged exposure to digital screens, air pollution, and an aging population [12]. 

DED is closely linked to systemic ailments, particularly autoimmune conditions [13-16]. These immune-mediated rheumatic disorders, a category marked by an excessively active immune system, encompasses conditions like Sjögren's syndrome (SS), rheumatoid arthritis (RA), systemic lupus erythematosus (SLE), systemic sclerosis (SSc), or scleroderma [13,14,17,18]. Beyond its connection to autoimmune diseases, DED is also correlated with ocular conditions like uveitis [15,19].

Autoimmune diseases affect multiple organs, resulting in diverse clinical symptoms such as skin lesions, joint pain, vascular damage, and dry eye [17,20,21]. Under normal circumstances, the immune mechanisms within the eyes maintain a balanced microenvironment. However, when the immune response in autoimmune diseases targets the eyes, it leads to excessive stimulation of the ocular immune system by specific immunoregulatory molecules, disrupting the balance of immune regulation [1,13,22]. Consequently, chronic inflammation on the ocular surface occurs due to an imbalance between the innate and adaptive immune systems, resulting in chronic dry eye [23].

Previous studies have predominantly focused on SS, as it presents with dry eyes and dry mouth, two common symptoms that have led researchers to explore the connection between dry eye and autoimmune diseases [14,23]. Unfortunately, a significant proportion of patients with dry eye with autoimmune diseases remain undiagnosed and untreated. Although the association between DED and autoimmune diseases has been known, the exact role of the latter in the disease process, its temporal evolution, and the processes involved at the cellular and molecular levels are still being elucidated. It is understood that inflammation is both a cause and a consequence of DED. This review article explores current knowledge in this area by summarizing the pathogenesis and the mechanism of DED that is related to autoimmune diseases, particularly focusing on SS, RA, SLE, SSc, sarcoidosis, and ocular rosacea. 

## Review

Historical timeline and current classification of DED

In 2007, TFOS DEWS released a comprehensive report that broadened our understanding of DED. Since then, the number of publications on DED has significantly increased. Nearly 10 years later, the much-anticipated TFOS DEWS II report was published online in a well-known journal [4-8,11,24-26]. This extensive report spans over 350 pages and serves as an update to the original TFOS DEWS, incorporating the latest research on DED. This newly updated article provided a brief overview of the key points from each section of the report, aiming to raise awareness about the significant findings and recommendations presented in this influential publication.

The main classification systems that are widely used are TFOS DEWS II and the Asia Dry Eye Society (ADES) [27]. TFOS DEWS initially divided DED into two main groups: aqueous-deficient and evaporative [28]. Aqueous deficiency is attributed to acinar ductal epithelial dysfunction, abnormal tear film composition, and low volume [28]. The evaporative group is highly attributed to meibomian gland dysfunction (MGD) and abnormal lipid layer composition [4]. However, since the revisions on report II, new diagnostic algorithm triage questions have been added. The TFOS DEWS II report proposed classification not as subgroups but rather as a broad spectrum [4]. Additional questionnaires, environmental risk factors, and symptom severity charts have been integrated into the algorithm as well [4,24]. In the latest published studies, DED subgroups have been revised and it has been underlined that it is harder to produce subgroups of DED as either ADDE or EDE [2,4,25]. This is mainly because the new proposed model suggests that these two exist in a continuum, rather than as two separate entities. 

Furthermore, ADES proposed an additional subgroup compared to TFOS, which classifies SS, an autoimmune disorder, separately [28]. Although neither has a set classification for autoimmune disorders, SS has been mentioned separately in ADES.

In the Western world, the primary instigator of ADDE is the inflammatory infiltration of the lacrimal gland, a phenomenon most pronounced in cases of DED linked to autoimmune disorders like SS. Additionally, albeit to a lesser extent, non-SS cases also exhibit this inflammatory infiltration [2,29]. Meanwhile, MGD, intricately associated with EDE, has been implicated in prior research. Shimazaki et al. illustrated that the substantial alterations in the ocular surface observed in SS patients can, at least partially, be ascribed to MGD [30]. MGD can exacerbate the symptoms of DED since the mechanism illustrates decreased oil secretion and unstable tear film production.

Epidemiology of autoimmune disease-related DED

According to previous studies and reports, autoimmune diseases are significant contributors to the occurrence of DED. DED is found in approximately 10-95% of patients with immunity-related disorders; 38-47% of individuals with RA experience DED, while the percentage is 13.4-39.5% for SLE, 95% for SS, and 37-79% for SSc [13,14,18,22,31-33]. Consequently, the prevalence of DED is higher in patients with SS compared to other autoimmune diseases. The proportion of females with autoimmune diseases is significantly higher than that of males [34]. Therefore, female patients are more likely to be affected by DED associated with autoimmune diseases.

The connection between SS and DED has been highlighted in guidelines published by the American Academy of Ophthalmology. These guidelines indicate that around 10% of individuals with DED also have SS [35]. A randomized controlled study involving 535 patients with moderate to severe DED revealed a significant association between SS and more severe dry eye symptoms [36]. Furthermore, the guidelines indicate that RA poses a risk factor for DED [35]. Likewise, Paulsen et al. proposed a close correlation between arthritis and symptoms of DED [37]. In a recent prospective multicenter study involving over 400 primary SS patients, DED symptoms were evident in 94% of cases [38]. Abd-Allah et al. discovered that RA can induce severe DED symptoms even in the absence of secondary SS [39]. Similarly, the occurrence of DED in SLE patients was significantly higher compared to individuals without the condition [40].

Pathogenesis and treatment options for dry eye and autoimmune disease-related DED

Patients with autoimmune diseases experience impaired and weak immune systems, leading to abnormal immune responses and disrupted immune regulation. This impairment puts the lacrimal gland, conjunctival membrane, cornea, and meibomian glands at high risk, causing tissue damage and dysfunction [7,41]. In individuals with autoimmune diseases, a notable proportion of immune cells, predominantly T lymphocytes, infiltrate the lacrimal duct and accessory lacrimal gland. This infiltration leads to autophagy and apoptosis of acinar, ductal, and myoepithelial cells [7,41]. This disrupts the function of the lacrimal gland and reduces tear secretion.

Abnormal antibodies trigger chronic fibrosis of the lacrimal gland and worsen its dysfunction due to the presence of innate immune cells and the secretion of inflammatory response factors. The infiltration of T cells in conjunctival tissue leads to squamous metaplasia, reduced goblet cell density, and decreased mucin secretion [42]. Furthermore, the deposition of autoantibodies and antigen-antibody complexes in corneal tissues causes corneal dystrophy [42]. Examination through confocal microscopy discloses an elevated count of corneal dendritic cells, coupled with diminished nerve density and sensitivity. Research indicates that individuals with SS-related DED exhibit significantly lower corneal nerve counts and increased tortuosity compared to the control group, consistent with earlier studies [43,44]. Furthermore, corneal nerve sensitivity experiences a significant reduction in patients with SS-related DED. In autoimmune diseases, there is a common occurrence of immune cell infiltration in the meibomian glands, leading to the mechanical obstruction of their ducts, thereby affecting the production of meibum [43]. Meibomian gland imaging can reveal atrophy and occlusion of certain ducts [45]. Moon et al. recently showed the linked gut dysbiosis to DED caused by autoimmune conditions like SS [46].

Regarding genetic heredity, RA is a complex disease associated with multiple genetic loci, each having a modest association with a specific condition. A recent study identified 39 variants in immune-related genes across families affected by SLE, RA, and SS [47,48]. Notably, these variants were concentrated in the regulation of T cell activation and T cell receptor signaling pathways, which may be related to DED. Consequently, genetic-related research proves to be a valuable tool in understanding the development of DED in autoimmune diseases. 

Treatment options for DED range anywhere from eliminating the risk factors and environmental factors to surgical approaches. The same approach also applies to autoimmune disease-related DED. Symptomatic relief has always been an important part to focus on for DED. Therefore, over-the-counter (OTC) medications are still widely utilized, in addition to topical medications. OTC artificial tears, lid hygiene products, and warm-compress are long-term favorites; adequate hydration as well as essential fatty acid supplementation are also recommended by TFOS guidelines as well as physicians. Artificial tears are beneficial for both ADDE by increasing the volume, as well as EDE. Topical cyclosporine application is also commonly used due to its anti-inflammatıry properties, seeing that inflammation plays a crucial part in DED, even though long-term uses may yield side effects. Another up-and-coming medication that has been named is lifitegrast, which plays a role in inhibiting inflammatory cell binding. Topical corticosteroids for a limited duration prescribed by the physician or topical secretagogues are within the scope of prescribed medications.

Overnight treatments such as moisture chambers and goggles are used in the comfort of patients' homes, and in addition to overnight treatments, office treatment options are available. In-office treatment options such as LipiFlow® (Johnson & Johnson, New Brunswick, New Jersey, United States) are recommended in cases of MGD. Gland expression through the LipiFlow system using heat is specifically recommended for patients who have EDE. Intense pulse light (IPL) uses a similar technology, where heat promotes the improved flow of meibum. In addition to alleviating DED symptoms, in cases where a patient has rosacea, IPL is also beneficial for getting rid of eyelid telangiectasias [49,50]. 

If the above recommendations are not found adequate for managing the symptoms and/or do not provide relief, scleral lenses for therapeutic purposes can be used and other minimally invasive approaches or surgical approaches may be recommended.

Selected autoimmune diseases-related DED

SS and DED

Primary SS is a complex systemic autoimmune disorder that mainly affects exocrine glands, including the salivary and lacrimal glands. Primary SS is most closely related to dry eye among the various types of autoimmune rheumatic diseases (ARDs)[36]. Destruction of the exocrine glands and lymphocyte infiltration plays an important role in pathogenesis in SS. SS typically presents in clinic visits with dry eye and dry mouth symptoms. It has been underlined that not only ocular discomfort but also visual disturbances are often described by the patients. As mentioned previously, in a recent prospective multicenter study with over 400 primary SS patients, 94% of the cases exhibited DED symptoms [38]. 

In patients with SS, activated T-cells target the lacrimal glands resulting in cell death and hyposecretion of tears, leading to ADDE; however, MGD may also be involved as well. [50] This leads us to an understanding that EDE also plays an important role in SS. When meibomian glands fail to produce the oil layer that overlays the tear film, the evaporation of the aqueous layer accelerates, resulting in EDE. Beyond the aqueous and oil layers, goblet cells in the conjunctiva produce mucins, constituting a third crucial component of tears. These mucins form the muco-aqueous layer of tears and play a role in stabilizing the tear film on the ocular surface [51]. It has been also underlined that an unstable tear film is more prone to evaporation, hence exasperating the symptoms of DED [32]. 

Even though treatment options for DED have been a topic of interest for many years, in the case of SS-related DED, the initial approach is to use artificial tears to supplement the reduced tear volume to decrease the friction between the lid and the globe. Due to the inflammatory processes that contribute to the progression, corticosteroid treatments as well as topical cyclosporine are within the treatment options. For patients with MGD, manual expression of meibomian glands, warming treatments such as warmed gel masks over eyelids, and continuous controlled thermal compression devices (LipiFlow system) are also considered for both symptomatic and therapeutic relief.

RA and DED

RA is categorized as an inflammatory, chronic autoimmune ailment primarily impacting synovial joints, as well as the skin, blood vessels, and eyes. The engagement of vascular, dermal, neural, and ocular tissues underscores RA as a multi-system disorder. The diagnosis of RA is established when a patient presents with inflammatory arthritis affecting three or more joints, positive rheumatoid factor (RF) and/or anti-citrullinated peptide/protein antibody (ACPA), a disease duration exceeding six weeks, and elevated levels of C-reactive protein (CRP) or erythrocyte sedimentation rate (ESR) [52]. It is important to note that where RF positivity is a part of the criteria of RA diagnosis, based on the 2010 American College of Rheumatology (ACR) /European League Against Rheumatism (EULAR) Diagnostic Criteria for RA, the terms of low positive RF and high positive RF in relation with ACPA yields to different points, which ultimately changes the score-based system when it comes to giving a definitive diagnosis [53]. However, any patient who has prolonged swelling in their joints without any other medical diagnosis should be considered for RA.

Previous retrospective studies observed that RA patients can develop secondary SS, which is common with DED patients [13,16,18]. Fujito et al. concluded that DED is common even in RA patients without SS [18]. Abd-Allah et al. found that RA can cause severe DED symptoms even in the absence of secondary SS [39]. The underlying mechanisms that lead to DED in both SS and non-SS patients with RA are different. Shan et al. highlight the importance of how inflammatory mechanisms play an important role in RA-associated KCS [16]. Another important point to keep in mind is how RA-related dry eye includes both aqueous-deficient and evaporative types of DED, affecting the conjunctiva and meibomian glands. Usuba et al. demonstrated the evaporative component disturbance in RA patients, with the presence of MGD (associated with alterations in the lipid layer of tear film) as well as decreased goblet cell numbers (associated with the mucin layer of the tear film), in addition to the traditional aqueous tear deficiency related to DED [16].

In the case of extra-articular manifestations of RA, ocular manifestations include episcleritis, scleritis, peripheral ulcerative keratitis (PUK), and DED, as seen in Table 1 [54]. Treatment modalities between episcleritis and scleritis differ. Episcleritis may be managed by symptomatic relief alone, while episcleritis often requires both topical and oral medication. The treatment of symptomatic episcleritis involves the local application of corticosteroid eye drops to accelerate resolution and alleviate bothersome symptoms; although episcleritis can resolve spontaneously, albeit more slowly, without treatment [55]. Nonsteroidal anti-inflammatory drugs (NSAIDs) are recommended to alleviate symptoms, and their inclusion in the treatment plan for episcleritis depends on the physician's expertise and preference [22]. Overall, retrospective studies suggest the use of NSAIDs or corticosteroids [13,55]. Systemic corticosteroids are extensively utilized and stand as the primary treatment for scleritis associated with RA [55]. Additionally, for RA-related scleritis, there is a broad indication for immunosuppressants [16,22,55]. These may serve as a third-step treatment or as part of combination therapy, especially in cases of necrotizing forms of rheumatoid scleritis. Commonly employed immunosuppressants include cyclophosphamide or methotrexate, alongside systemic corticosteroids. Prednisone is the preferred corticosteroid, with an initial dose of 1 mg/kg per day prednisone equivalent, sometimes preceded by intravenous boluses of methylprednisolone (1 g per day for three days) in instances of vision-threatening complications [13,16,55]. In cases with RA-associated PUK, if the initial treatment module with systemic corticosteroids and methotrexate is not sufficient to promote epithelial healing, it is recommended to use cyclophosphamide and azathioprine as an alternative for methotrexate. A beneficial effect of combining corticosteroids and cyclophosphamide for the treatment of PUK associated with RA has been indicated by evidence from several retrospective studies [46,56].

**Table 1:** Select autoimmune diseases and their related ocular manifestations KCS: keratoconjunctivitis sicca; PUK: peripheral ulcerative keratitis; DED: dry eye disease

	Sjögren Syndrome	Rheumatoid Arthritis	Systemic Lupus Erythematosus	Sarcoidosis	Systemic Sclerosis	Rosacea
Incidence of DED	95%	20-25%	13.4-39.5%	42-63%	37-79%	56-72%
More commonly affected gender	Female	Female	Female	M = F	Female	Female
Symptom onset according to age (years)	40-60 (58 mean age according to article)	40-60	20-30	20-40	30-60	20-30
Most common ocular manifestation	Dry Eye Symptoms	Dry Eye Symptoms	Dry Eye Symptoms	Uveitis, Bilateral Anterior Uveitis	Dry Eye Symptoms	DED + Blepharitis and MGD
Other ocular manifestations	Visual disturbances	KCS, episcleritis, scleritis, PUK	KCS, episcleritis, scleritis, PUK	KCS, conjunctivitis, conjunctival nodules, lacrimal gland enlargement	Conjunctivitis, episcleritis, anterior uveitis, hypertensive retinopathy	DED, chronic conjunctivitis, superficial punctate keratitis, iritis, episcleritis, and scleritis
Type of dry eye	Aqueous deficent (main); also, evaporative due to MGD	Evaporative	Evaporative and Aqueous Deficient	Evaporative	Aqueous Deficient	Evaporative
Main reason identified for DED	Lymphoid infiltration of lacrimal gland, chronic inflammation of lacrimal gland	Inflammation of meimoban gland	Chronic inflammation of meimobian gland and lacrimal gland + secondary Sjögren syndrome	Lacrimal gland inflammation	Fibrosis of conjunctiva and lacrimal glands	Inflammation of MGD

SLE and DED

SLE is a chronic autoimmune disease that affects multiple systems in the body [22]. It is more prevalent in women, with a female-to-male ratio of 6:1 to 10:1, and typically affects women of childbearing age [31]. Approximately one-third of SLE patients experience ocular symptoms, and the incidence of SS as a secondary condition to SLE is about 13.96% [22]. Dry eye symptoms are often overlooked in SLE patients without secondary SS, even though dry eye is the most common ocular manifestation of SLE, including juvenile SLE [13].

DED is an important indicator of SLE and is diagnosed in different stages of the disease: 5% in early-onset SLE, 16% in the duration stage, and 33% in late-onset SLE [13]. To predict flare-ups and assess disease activity in SLE, elevated levels of anti-double-stranded DNA antibodies (anti-dsDNA) and erythrocyte sedimentation rate, as well as decreased complement levels (C3 and C4), are commonly used [22]. Researchers have found that in SLE patients without secondary SS, the severity of dry eye correlates with anti-dsDNA titers and low C3 levels, but not with low C4 levels, erythrocyte sedimentation rate, and antinuclear antibodies [31].

SLE not only impacts the density and morphology of corneal Langerhans cells but also disturbs corneal homeostasis, potentially playing a role in the onset of dry eye [14]. Impaired meibomian gland function and compromised tear film lipid layers emerge as notable risk factors for dry eye in SLE patients without secondary SS [14]. SLE can influence various components associated with DED, including the lacrimal gland, cornea, and meibomian glands, as summarized in Table 1 [13].

Treatment strategies for SLE include non-steroidal anti-inflammatory drugs, hydroxychloroquine, systemic corticosteroids, immunosuppressive therapy, and biologics [13]. The effective immunosuppressive drugs include azathioprine, methotrexate, mycophenolate mofetil, and cyclophosphamide. Hydroxychloroquine is an effective medication for SLE. It is now recommended long-term for all patients with SLE [57].

Sarcoidosis and *DED*

Sarcoidosis is one of the leading causes of inflammatory eye disease [58]. It commonly affects the lacrimal gland in the orbit, with previous histopathological studies showing involvement in 42-63% of cases [58,59]. Enlargement of the lacrimal gland can result in palpable masses or other symptoms due to the pressure exerted by the mass [60]. The most common ocular manifestation overall is uveitis, reported in 30-70% of cases [61]. Sarcoidosis can present with anterior, intermediate, posterior, or panuveitis and should be on the differential for any patient with uveitis [62]. The most common type is bilateral anterior uveitis without posterior-segment involvement, which is summarized in Table 1 [62].

In addition to uveitis, DED is a common consequence of sarcoidosis [63,64]. It can cause symptoms such as irritation, tearing, corneal epitheliopathy, corneal abrasion, infection, and permanent corneal scarring. In case of complications arising from both chronic uveitis and DED symptoms, cataract formation, and glaucoma may lead to vision loss [62]. DED is commonly associated with sarcoidosis and is a result of decreased aqueous tear production secondary to lacrimal gland inflammation [62]. 

Previous studies have documented abnormalities in imaging and biopsy results of the main lacrimal gland, with specific mention of the accessory lacrimal glands lacking in some cases [60,63,64]. However, there have been documented reports of the involvement of full-thickness eyelid skin [64]. Physiologically, the main lacrimal glands situated in the anterolateral part of the orbit are responsible for reflex tear secretion, while the smaller accessory lacrimal glands in the eyelids and conjunctiva primarily produce basal tears [65].

For anterior uveitis, topical corticosteroids serve as the first-line treatment but may prove ineffective against posterior segment inflammation [61]. Systemic steroids may be employed in cases of chronic progression of ocular symptoms as part of an overall treatment regimen. However, due to potential systemic side effects, it is recommended to use the lowest effective dose in treatment modalities [61]. Immunosuppressive agents like methotrexate, azathioprine, and cyclosporine are commonly utilized, although the choice may vary among physicians.

SSc and DED

SSc or scleroderma is a multifaceted autoimmune disease with an unknown cause [13,33]. It is characterized by tissue fibrosis and vasculopathy affecting the skin and various organs [17]. The development, severity, and onset of the disease are influenced by both genetic and environmental factors [13]. The highest occurrence of systemic sclerosis is observed in individuals aged between 30 and 60 years, with a higher prevalence among females compared to their male counterparts [33]. However, men tend to experience a more severe form of the disease, including complications in internal organs and increased mortality rates [33]. The most common ocular manifestation of SSc is DED followed by conjunctivitis, episcleritis, anterior uveitis, and hypertensive retinopathy [33,66,67] (Table 1).

The clinical symptoms of the condition arise from three separate mechanisms: abnormalities in the innate and adaptive immune systems, which result in the production of autoantibodies and cell-mediated autoimmunity, vasculopathy affecting small blood vessels, and dysfunction of fibroblasts causing an excessive buildup of collagen and other matrix components in the skin, blood vessels, and internal organs [13,33]. 

DED in SSc is likely caused by fibrosis of the conjunctiva and lacrimal glands [33]. The mechanism of DED in primary SS and SSc is similar because both involve the primary lacrimal gland duct [13,33]. However, lymphoid infiltration in the gland is less pronounced in SSc, which helps differentiate SSc-related DED from SS-related DED [13]. The presence of fibrosis indicates SSc, while lymphocytic infiltration is a key indicator of primary SS [68]. Peripheral neuropathy, commonly associated with SSc, may lead to decreased corneal sensation in patients, which could explain the lack of correlation between dry eye signs and symptoms [13]. Blepharitis and MGD are also contributing factors to SSc-related DED [69]. The lacrimal gland, conjunctiva, cornea, and meibomian glands are all involved in SSc-related DED [33,69]. 

While there is no cure for SSc, there are treatment options to alleviate symptoms of SSc-related DED. The use of lubricating eye drops or artificial tears may help ease the DED symptoms. Furthermore, eyelid hygiene is another important part that can help maintain ocular surface hemostasis in patients with DED to SSc [70]. The use of contact lenses can help trap the moisture in the eyes, which can be beneficial to ease the symptoms [33]. 

Ocular rosacea and DED

Rosacea is a long-lasting inflammatory skin condition that causes redness, visible blood vessels, small bumps, and pus-filled pimples on the face, primarily in the central area [71,72]. It is a prevalent condition affecting around 14 million individuals in the United States [73]. The onset of rosacea usually occurs between the ages of 20 and 30 years, and it becomes more apparent in the following 10 years [73]. Although considered a skin disease, rosacea may evolve the eyes in up to 58-72% of the patients, causing eyelid and ocular surface inflammation [74-76]. About one-third of the patients develop corneal involvement, which may be sight-threatening [74-76]

Ocular rosacea can impact the corneal surface, especially in the presence of dry eyes resulting from tear evaporation [71]. During ocular clinical examinations, various signs are evident, including flaky skin, dilated blood vessels at the eyelid edges, reduced tear film stability, clogged meibomian glands, conjunctival redness, small corneal damage areas, the formation of new blood vessels in the cornea, and the development of ulcers (refer to Table 1) [71,77]. Ocular rosacea may contribute to or exacerbate MGD, intensifying DED symptoms. Dry eyes, confirmed by an abnormal Schirmer test, were reported in 56-62.5% of patients with ocular rosacea [78,79]. A reduced tear break-up time (TBUT) has also been documented in a substantial majority of individuals with ocular rosacea [71,78,79]. Additionally, blepharitis and MGD are common findings alongside DED [33,80].

Previous studies have shown that oxytetracycline has a moderate therapeutic effect on ocular rosacea [71,81]. Since inflammation plays a role in ocular rosacea, topical steroid eye drops have been reported to effectively prevent recurrent corneal erosion associated with ocular rosacea when used in combination with oral doxycycline [81]. Although not specifically evaluated for ocular rosacea, RESTASIS® (ophthalmic cyclosporine) possesses anti-inflammatory properties, which are useful for DED treatment, that may be beneficial in treating the dry eye symptoms associated with rosacea as well [81]. As mentioned previously, IPL has been used to alleviate DED symptoms in patients with rosacea [49]. 

## Conclusions

DED is the most prevalent ocular complication observed in autoimmune diseases. In autoimmune disease-related DED, various functional abnormalities affect the lacrimal gland, lacrimal duct, conjunctiva, cornea, and meibomian glands, resulting in both ADDE and EDE occurring simultaneously. Patients with autoimmune diseases often experience severe dry eye symptoms, making it a noteworthy indicator of these conditions. Exploring autoimmune disease-related dry eye in future research could provide insights into the systemic immunological and molecular mechanisms involved in autoimmune diseases. It is crucial to develop a comprehensive understanding of DED, autoimmune diseases, and their associated complications.
